# Clozapine induced pancytopenia leading to severe sepsis: an unusual early complication

**DOI:** 10.1186/s13104-015-1777-5

**Published:** 2015-12-16

**Authors:** Jagath Pushpakumara, Piyumanthi Karunarathna, Sivagamaroobasunthari Sivathiran, Ajantha Liyanage, Jegarajah Indrakumar

**Affiliations:** Ward 01, Department of Medicine, University Medical Unit, Colombo South Teaching Hospital, Kalubowila, Sri Lanka; Department of Medicine, Faculty of Medical Sciences, University of Sri Jayewardenepura, Gangodawila, Nugegoda, Sri Lanka

**Keywords:** Clozapine, Pancytopenia

## Abstract

**Background:**

Clozapine is a second generation antipsychotic used to treat resistant schizophrenia and other psychotic illnesses. Leucopenia or agranulocytosis is a rare side effect of this drug. Pancytopenia is an extremely rare side effect of clozapine and literature review showed only one such case in where the pancytopenia developed several months after starting clozapine together with other antipsychotic drugs.

**Case presentation:**

A 26-year-old Sri Lankan male was admitted with fever for 3 days. Apart from generalized body aches there were no other significant symptoms. His blood counts showed pancytopenia. He was being treated for a resistant schizophrenia and clozapine was started only 4 weeks before. Common causes for pancytopenia were excluded, and a diagnosis of clozapine induced pancytopenia was made. He was managed in the intensive care unit with broad spectrum antibiotics, antifungals and granulocyte colony stimulating factors. He made a complete recovery after 4 weeks.

**Conclusion:**

This is a rare and probably the first reported case of early onset clozapine induced pancytopenia complicated by severe sepsis recovering completely.

## Background

Clozapine is an atypical antipsychotic drug. It relieves both negative and positive symptoms of schizophrenia and is the drug of choice in resistant schizophrenia [1, 2]. Clozapine causes leucopenia or agranulocytosis as a rare side effect. Clozapine induced pancytopenia is even rarer than leucopenia and review of literature showed only one published case. Agranulocytosis is defined as granulocyte count of <0.5 × 10^3^/µl, and leukopenia is defined as white blood cell (WBC) count of <3.5 × 10^3^/µl. The causes for the blood dyscrasias are believed to be due to idiosyncratic (type B) reactions, immune-mediated or toxic suppression of hemopoietic precursors due to prolonged administration [2]. In our patient early occurrence of pancytopenia is most likely due to an idiosyncratic drug reaction.

## Case presentation

A 26-year-old Sri Lankan male was admitted to hospital with fever for 3 days and body aches. He did not have any other significant symptoms. He was diagnosed of having schizophrenia 4 years ago and was attending a psychiatric clinic. He was started on clozapine, 4 weeks ago (200 mg daily), because of inadequate control of symptoms with conventional antipsychotics. On examination he was febrile (102 °F), not pale or icteric. He did not have lymphadenopathy. Blood pressure was 130/80 mmHg and pulse rate was 110 bpm with good volume. His respiratory rate was 32 cycles per minute. Examination of the abdomen, cardiovascular and respiratory systems were unremarkable.

His full blood count (FBC), which was done a day prior to hospital admission showed white blood cell count (WBC) 2.9 × 10^3^/µl, absolute neutrophil count 667/µl (23 %), lymphocytes 2117/µl (73 %), Hemoglobin (Hb) 11.3 g/dl, platelet 188 × 10^3^/µl. The first FBC during the hospital stay showed reduced blood counts, of which WBC 0.2 × 10^3^/µl with absolute neutrophil count (ANC) 110/µl, lymphocyte count 80/µl, Hb 12.2 g/dl and platelet 972 × 10^3^/µl. The C reactive protein was (CRP) 270 mg/L (<6 mg/L). His chest X ray, ultrasound scan (USS) of abdomen and pelvis, urine full report was normal. Serology for dengue virus, human immunodeficiency virus and parvo B19 virus were negative. Electrocardiogram (ECG) showed sinus tachycardia.

**Fig. 1:**
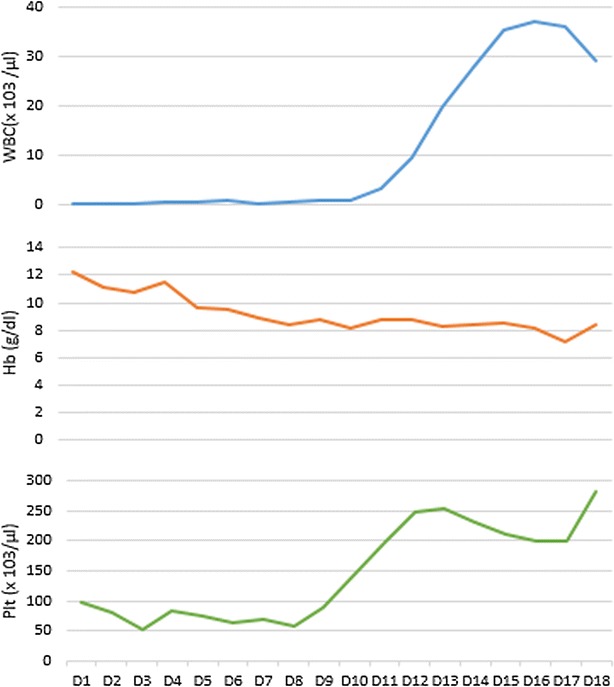
Demonstration of pancytopenia and its recovery with the treatment

Clinical diagnosis of neutropenic sepsis was made. He was treated with intravenous imipenum, vancomycin, clindamycin, metronidazole and fluconazole. His serial blood cultures, urine cultures and sputum cultures were negative. His FBC was monitored twice daily throughout hospital stay, showed severe neutropenia and thrombocytopenia which later complicated by anemia from the 3rd day of hospital stay (Table 1; Fig. 1). The reticulocyte count was 1.1 % (normal 0.5–2.5). The patient was transferred to intensive care unit (ICU) on the 3rd day and treated with granulocyte colony stimulating factors (G-CSF) 300 µg daily for 10 days. On the 5th day he developed swelling of his left inguinal region with mild skin induration. The initial USS of left groin showed only subcutaneous tissue oedema. This subsequently progressed to an abscess which was drained. His white cell count and platelet count improved with 10 days of treatment with G-CSF. Hemoglobin normalized in 4 weeks of treatment. At this stage his WBC count was 12 × 10^3^/µl with absolute neutrophil count 7200/µl (60 %), hemoglobin 12.6 g/dl and platelets 212 × 10^3^/µl. He was started on conventional antipsychotics without clozapine for his psychiatric condition.

**Table 1:** Summary of the investigations during the first 18 days of hospital stay

Investigation	D1	D2	D3	D4	D5	D6	D7	D8	D9	D10	D11	D12	D13	D14	D15	D16	D17	D18
WBC (×10^3^/μl)	0.20	0.32	0.40	0.60	0.60	0.80	0.32	0.61	0.82	1.0	3.5	9.5	20	27.7	35.5	37.2	36.1	29.4
ANC (×10^3^/μl)	0.11	0.10	0.16	0.09	0.12	0.14	0.14	0.28	0.15	0.34	1.8	6.9	16.5	21.6	28	29.6	31	25
Hb (g/dl)	12.2	11.1	10.7	11.5	9.6	9.5	8.9	8.4	8.7	8.1	8.8	8.7	8.3	8.4	8.5	8.2	7.1	8.4
RBC (×10^6^/μl)	4.19	4.21	3.95	3.95	4.01	4.65	3.56	3.88	4.28	4.94	4.22	3.98	4.11	4.55	4.82	4.77	4.36	4.29
MCV (fL)	84	85	85.4	85.3	85.4	84	83.1	86	84.1	83.1	85	84.2	83.2	85	84.1	86	86.2	85.3
MCH (pg)	32.0	30.1	29.8	30	28.8	29.1	31	32.2	31.3	33	34	32.1	33.4	32	34	33.1	33	33.2
MCHC (g/dl)	34.9	35.1	34.9	35.2	33.7	34.4	35	35.2	33	34	33.2	34	33.2	35	35.2	34.8	33.8	32.7
PCV (%)	35	33	30	36	28	27	27	25	25	26	24	26	26	25	26	25	22	26
Plt (×10^3^/μl)	97	79	53	82	75	64	70	57	89	144	196	247	254	232	212	200	201	283
ALT (IU/l)		43	42			35	63	58	60	53	48	56	62	49	38	23		30
AST (IU/l)		103	114			67	108	70	65	68	74	80	68	52	50	65		42
Alb (g/l)						22			23			28				26		
Prot (g/l)						44			56			60				60		
S cr (μmol/l)		56.2				71	75	64	51	47			66			73		68
BU (mmol/l)	15.7	14.9			9.3	7.0	6.7	4.3	4.3	4.6	3.8	4.2	5.0	4.8	3.8	2.5		
Na^+^ (mmol/l)	134	132			140	147	148	154	132	153	143	140	138	132	136	145	138	136
K^+^ (mmol/l)	4.4	3.6			3.5	3.8	3.3	3.4	4.1	5.3	4.8	3.9	4.0	3.1	3.0	3.2	4.1	4.8
CRP (mg/l)				270						30								34

*WBC* white blood cells, *ANC* absolute neutrophil count, *Hb* hemoglobin, *RBC* red blood cell count, *MCV* mean corpuscular volume, *MCH* mean corpuscular hemoglobin, *MCHC* mean corpuscular hemoglobin concentration, *PCV* pack cell volume, *Plt* platelet, *ALT* alanine transaminase, *AST* aspartate transaminase, *Alb* albumin, *Prot* total protein, *S cr* serum creatinine, *BU* blood urea, *Na* ^+^ serum sodium, *K* ^+^ serum potassium, *CRP* C reactive protein

## Discussion

This case illustrates an extremely rare complication of pancytopenia caused by clozapine. Clozapine is known to cause agranulocytosis or neutropenia as a rare side effect. Approximately 2.7 % of patients treated with clozapine develop neutropenia, of which 0.9 % develops agranulocytosis [3]. Most cases (50–80 %) occur during 18 weeks after commencing the treatment [3–5].

Baseline FBC should be routinely done before commencing clozapine and total WBC should be more than 4 × 10^3^/µl with ANC more than 2.5 × 10^3^/µl [5, 6]. It is recommended that weekly FBC should be done to detect blood dyscrasia early. The patient’s WBC count, at the time of starting clozapine was 7.7 × 10^3^/µl with ANC 3927/µl (51 %), lymphocytes 3080/µl (40 %), eosinophils 462/µl (6 %), and platelets 260 × 10^3^/µl. Hematological results are reported as ‘green’ (safe to continue treatment), ‘amber’ (borderline low or falling result) and ‘red’ (below the acceptable reference range). If the WBC subsequently drops below the ‘red’ cut-off of 3 × 10^3^/µl or the neutrophil count falls below 1.5 × 10^3^/µl, clozapine treatment must be stopped [6]. In this patient pancytopenia was detected at 4 weeks of clozapine treatment and there were no blood counts done during this period.

There was only one reported case of 57-year-old male with clozapine induced pancytopenia who was subsequently managed with electroconvulsive therapy (ECT) [7]. This patient was on clozapine for several years which was later combined with fluphenazine and promazine. He developed neutropenia after 1 year of combined antipsychotics therapy. Clozapine was stopped and restarted after 1 year and the patient had developed pancytopenia after about one and half year of therapy. His blood counts had spontaneously improved within 7 weeks after stopping clozapine. However In our patient the pancytopenia occurred within the first 4 weeks of treatment initiated with with clozapine.

Stübner et al. described the blood dyscrasias induced by psychotropic drugs after analyzing 122,562 patients in 35 psychiatric institutions since 1993–2000 [8]. In this study, 107 cases of hematological changes had been documented with an incidence of 0.0873 %. Of these, pancytopenia was seen only in 2 and rest were neutropenia (63 cases), thrombocytopenia (16 cases), agranulocytosis (22 cases) and neutropenia thrombocytopenia (4 cases). Of these 2 cases of pancytopenia, both carbamazepine and lovastatin were rated as probable causative agents in combination in one case. In the other case, pancytopenia was observed during treatment with trimipramine. Clozapine induced pancytopenia was not observed in this study.

Recovery of blood dyscrasia usually occurs up to 4 weeks after clozapine withdrawal, but there were exceptions—in one case report thrombocytopenia and leucopenia both occurred during clozapine treatment and persisted for 13 weeks [7]. Our patient recovered successfully from clozapine induced pancytopenia within 3 weeks. We emphasize that every patient who is started on clozapine need very close monitoring of FBC to detect blood dyscrasia to minimize serious complications.

## Conclusion

Clozapine induced pancytopenia is extremely rare and periodic FBC should be done to detect blood dyscrasia early. We report an unusual and early complication of clozapine induced pancytopenia leading to severe sepsis recovered with antibiotics, granulocyte colony stimulating factors and supportive care.

## Consent

Written informed consent was obtained from the patient for publication of this case report and any accompanying images.

## Abbreviations

FBC: full blood count
WBC: white blood cell count
ANC: absolute neutrophil count
CRP: C reactive protein
USS: ultra sound scan
ECG: electrocardiogram
G-CSF: granulocyte colony stimulating factors
ICU: intensive care unit
ECT: electroconvulsive therapy
